# Therapeutic effect of modified zengye decoction on primary Sjogren’s syndrome and its effect on plasma exosomal proteins

**DOI:** 10.3389/fphar.2022.930638

**Published:** 2022-08-26

**Authors:** Yimin Zeng, Xin Peng, Yun Wang, Lei Hou, Wukai Ma, Peng Yang

**Affiliations:** The Second Affiliated Hospital, Guizhou University of Traditional Chinese Medicine, Guiyang, China

**Keywords:** Sjögren’s syndrome, exosomes, proteomic, efficacy evaluation, modified zengye decoction

## Abstract

**Background:** Modified Zengye Decoction (MZD), a traditional Chinese medicine, is an effective treatment for patients with primary Sjögren’s syndrome (pSS).

**Purpose:** To evaluate the efficacy of MZD and investigate its effect on plasma exosomal proteins.

**Methods:** Eighteen pSS patients were treated with MZD for 2 weeks. The therapeutic effect was evaluated by observing the changes in clinical symptoms, laboratory parameters**,** and plasma cytokines before and after treatment. Then, the differentially expressed proteins (DEPs) in the plasma exosomes before and after treatment were identified via label-free proteomics, while Gene Ontology (GO) and Kyoto Encyclopedia of Genes and Genomes (KEGG) enrichment were used to analyze the possible biological functions and signaling pathways involved in the exosomal DEPs.

**Results:** MZD can effectively relieve the clinical symptoms of pSS patients, downregulate the plasma IgG and IgM levels, and inhibit plasma cytokine production. Thirteen DEPs were identified via label-free proteomics in the plasma exosomes before and after MZD treatment, of which 12 were downregulated proteins. GO analysis showed that these downregulated proteins were mainly related to the insulin response involved in dryness symptoms and the Gram-negative bacterial defense response and proteoglycan binding involved in infection. KEGG enrichment analysis showed that these downregulated proteins were primarily associated with the porphyrin metabolism involved in oteoarthrosis and the NF-κB and TLR4 pathways involved in infection.

**Conclusion:** MZD can effectively alleviate SS symptoms, while its mechanism may be associated with the reduced protein expression in insulin response, porphyrin metabolism, and the TLR4/NF-κB pathway.

## 1 Introduction

Primary Sjögren’s syndrome (pSS) is an autoimmune disease caused by damage to the exocrine gland tissue, characterized by dry eyes and a dry mouth. Viral infections and hormonal factors cause abnormal epithelial cell immune function in genetically susceptible hosts, recruiting T cells, B cells, and dendritic cells that target the diseased tissue (Arvaniti et al., 2019; Maślińska, 2019). This induces the immune system to produce an immune response against its own exocrine gland epithelial cells, generating many autoantibodies and cytokines, resulting in the destruction and dysfunction of local exocrine gland tissue, while multiple systems and tissue lesions may be involved in severe cases (Rivière et al., 2020). The current treatment for the disease remains largely palliative, focusing on stimulating the secretion of the remaining exocrine glands or replacing the exocrine fluid of saliva and tears (Chowdhury et al., 2021; Fox et al., 2021). However, the existing treatments are primarily unsuccessful in improving symptoms and disease progression.

Traditional Chinese medicine (TCM) typically consists of various herbs to treat multiple conditions (Ma et al., 2016; Lu et al., 2021). It is beneficial for treating multi-system and multi-tissue diseases (Yuan et al., 2016; Shi et al., 2020). Zengye Decoction is a classic TCM prescription containing *Scrophularianingpoensis Hemsl* (Xuanshen), *Ophiopogonjaponicus* (Maidong) and *Rehmanniagutinosa* (Shengdihuang), which increase fluid and alleviates dryness. Zengye Decoction was first mentioned involume II of the classic medical"WenbingTiaoxi” and is mainly used to treat dryness, including constipation, thirst, and a dry tongue. Some reports showed that Zengye decoction or modified Zengye decoction could effectively alleviate SS symptoms (Wu et al., 2014; Jiang et al., 2021; Wei et al., 2021). According totraditional Chinese medicinal theory, the patients in this study displayed a “toxic accumulation and blood stasis” during the early SS stage. Therefore, this study added detoxifying *Artemisia carvifolia Buch* (Qinghao), *Oldenlandiadiffusa* (Baihuashecao), *Andrographispaniculata* (Chuanxinlian), and *Gnaphalium* affine (Shuqucao) during the clinical application of Zengye Decoction for SS treatment to obtain Modified Zengye Decoction (MZD). MZD displays detoxification properties by increasing moisture and alleviating dryness, increasing the therapeutic role of traditional Chinese medicine in treating SS patients. However, the specific therapeutic effect and mechanism of traditional Chinese medicine on SS remain unclear.

Exosomes are bilayer lipid membrane vesicles secreted by cells in the form of budding. Exosomes have specific markers such as CD9, CD63, and CD81 on their surfaces and contain a large number of protein, nucleic acid, and lipid molecules, which extensively participate in intercellular information transmission in a non-cellular contact manner (Gurunathan et al., 2019; Kalluri and LeBleu, 2020). Studies have shown that protein information molecules in exosomes regulate T and B lymphocyte activation (Anel et al., 2019)and are associated with the immune pathogenesis of autoimmune diseases (Tanet al., 2016), including rheumatoid arthritis (RA), Sjögren’s syndrome and systemic lupus erythematosus (SLE). Since many TCM compounds play a therapeutic role based on immune regulation (Ma et al., 2013; Wang et al., 2020), and it is reported that *Ophiopogon japonicus*, one of the main drugs constituting Zengye decoction, can effectively regulate the differentiation of T lymphocyte subsets and inhibit the production of auto-antibodies in SS patients (Wang et al., 2007), we speculate that the therapeutic effect of Zengye Decoction on pSSmay be related to the changes of content in blood exosome. This study evaluates the therapeutic impact of MZD by observing the changes in clinical symptoms, laboratory indicators, and cytokines before and after SS treatment and aims to clarify the possible action mechanism of MZD via plasma exosomal proteins using label-free proteomics and bioinformatics.

## 2 Methods

### 2.1 Study design

The participating pSS patients were treated with MZD for 2 weeks, and the changes in their clinical symptoms, laboratory indicators, and plasma cytokines were compared before and after treatment. Furthermore, the differentially expressed proteins (DEPs) in the plasma exosomes before and after MZD treatment were identified via label-free proteomics, while GO and KEGG enrichment were used to analyze the biological characteristics of the downregulated DEPs.

### 2.2 Patients

Sixty patients were recruited from the Second Affiliated Hospital of Guizhou University of Traditional Chinese Medicine to participate in this trial. Twenty-six patients with hypertension, hyperglycemia, hyperlipidemia, gout, rheumatoid arthritis, and hepatitis B viral infection were excluded, while 16 used hydroxychloroquine hydrochloride and other Western drugs during the treatment. The remaining 18 pSS patients met the requirements of this trial and the standard classification criteria of pSS jointly presented by the American College of Rheumatology (ACR) and the European League Against Rheumatism (EULAR) (Shiboski et al., 2017). This study required a patient score exceeding or equal to 4, without the use of Western or traditional Chinese medicine in the previous 2 weeks. All subjects signed an informed consent form after reviewing the details of the trial.

### 2.3 Modified zengye decoction formula and usage

The granule pharmacy of the Second Affiliated Hospital of Guizhou University of Traditional Chinese Medicine provided 40 g Qinghao, 20 g Shengdihuang, 20 g Xuansheng, 20 g Maidong, 20 g Baihuashecao, 20 g Chuanxinlian, and 10 g Shuqucao (4:2:2:2:2:2:1) in granulated form. Here, 100 ml of boiling water was added to 7.5 g of each sample and stirred to mix thoroughly. The patients took these mixtures 30 min after meals, twice a day for 2 weeks.

### 2.4 Plasma preparation

Venous blood treated with EDTA was used to prepare the plasma samples of the participants. These samples were centrifuged at 3,000 g for 30 min (4°C)to remove the cells and cell debris, after which the supernatant was stored at −80°C for downstream analysis.

### 2.5 Exosome isolation

The plasma exosomes were isolated using ultracentrifugation (UC)with some modifications (Diaz et al., 2018). Briefly, the plasma samples were thawed at 37°C and centrifuged at 500 g for 5 min and 2,000 g for 20 min at 4°C to eliminate the cells and cell debris. The samples were then subjected to UC at 10,000 g at 4°C for 45 min to remove larger vesicles. Next, the supernatant was passed through a 0.45-μm pore filter, followed by UC at 100,000 g at 4°C for 75 min. The supernatant was discarded, after which the pelleted exosomes were resuspended in 200 μl PBS, of which20 μL was used for electron microscopy and10 μl for particle size measurement, while the remaining exosomes were stored at −80°C.

### 2.6 Label-free quantitative proteomic analysis

#### 2.6.1 Preparation of the plasma exosome samples

The plasma exosomes of nine patients were divided into three random groups. Each group was pre-treated with a plasma exosome mixture from three patients, labeled pre-SS1, pre-SS2, and pre-SS3. Similarly, each group received post-treatment containing a mixture of the plasma exosomes from patients treated with MZD, labeled post-SS1, post-SS2, and post-SS3.

#### 2.6.2 Exosomal protein preparation

Briefly, the plasma samples of three pSS patients were centrifuged at 1,000 g for 10 min. The subsequent supernatant was centrifuged at 17,000 g for 15 min at 4°C, followed by UC at 4°C and 200,000 g for 1h. The supernatant was then discarded. For the 1 × PBS resuspended precipitation, urea particles were added until reaching a concentration of 8 M and shaken until fully dissolved. Ultrasonic crushing on ice, 30% energy, ultrasonic for 1 s, stop for 1 s, cumulative 2 min.The sample was centrifuged at 14,000 g for 20 min, and the supernatant was collected, 10 μl of which was used for protein quantification using a BCA protein assay kit, while the remainder was frozen at −80°C.

#### 2.6.3 Protein digestion and desalination

A 100-µg aliquot of extracted proteins from each sample was subjected to a reduction process. A200 mM dithiothreitol (DTT) solution was added and incubated at 37°C for 1 h. The samples were diluted four times by adding 25 mM ammonium bicarbonate (ABC) buffer. Next, trypsin (trypsin: protein = 1:50) was added and incubated overnight at 37°C, followed by the addition of 50 μl 0.1% FA to terminate the digestion process. Then, 100 μl of 100% CAN was used to wash the C18 column, followed by centrifugation at 1,200rpm for 3min. The column was washed once with 100 μL of 0.1% FA and centrifuged at 1,200 rpm for 3 min. After the EP tube was replaced, the sample was added and centrifuged at 1,200 rpm for 3 min. The column was washed twice with 100 μl of 0.1% FA, followed by centrifugation at 1,200 rpm for 3 min, and then washed with 100 μl water. The EP tube was replaced, followed by elution with 70% ACN. The eluents of each sample were combined, lyophilized, and stored at −80°C until loading.

#### 2.6.4 LC-MS/MS analysis

The mobile phase A (99.9% water and 0.1% formic acid) and B solutions (100% acetonitrile and 0.1% formic acid) were prepared. Lyophilized powder was dissolved in 10 μl of solution A and centrifuged at 14,000 g for 20 min at 4°C, after which 1 μg of the supernatant was injected into a C18 column. The peptides were separated via linear gradient elution and analyzed using a Q Exactive HF-X mass spectrometer (Thermo, Waltham, MA, United States) equipped with a Nanospray Flex™ (ESI) ion source at a spray voltage of 2.4 kV and an anion transport capillary temperature of 275°C. Full-scan mass spectrometry was performed in a range of 350 m/z to 1500 m/z at a resolution of 120,000 (at 200 m/z), an automatic gain control (AGC) target value of 3×10^6^, and a maximum ion injection time of 80 ms. The top 40 precursors of the highest abundance during the full scan were selected and fragmented via higher-energy collisional dissociation (HCD). The samples were analyzed *via* MS/MS at a resolution of 15,000 (at200 m/z), an AGC target value of 5 × 10^4^, a maximum ion injection time of 45 ms, and normalized collision energy of 27% (Cox et al., 2014).

#### 2.6.5 Data analysis

The raw MS data were analyzed using Proteome Discoverer2.4software and searched against the *Homo sapiens* database in the Universal Protein Resource Knowledge Base (UniProtKB). The precursor mass window was 15 ppm for the initial search and followed the enzymatic cleavage rule for trypsin. Amaximum of two missed cleavage sites and a 20-ppm mass tolerance for the fragment ions were allowed. The cutoff for the global false discovery rate (FDR) in the peptide-spectrum match (PSM) and protein identification was *p* < 0.01. The complete peptide and protein information from the triplicate analyses of the three samples is available in the ProteomeX change Consortium via the PRIDE partner repository with the dataset identifier, PXD010556. Only proteins identified by at least two different peptides and in at least two sample replicates were regarded as present, reliably identified, and subjected to further analysis (Yan et al., 2011).

#### 2.6.6 Protein quantification and data processing

The label-free quantification (LFQ) of each identified protein was performed using the peptide signal intensities. The MaxLFQ algorithm in MaxQuant was activated to quantify the protein abundance of the identified peptides. Match-between-runs were performed during the experimental replicates to extract the comprehensive quantification information. Perseus was used to complement a value from the normal distribution with default parameters for low-abundance proteins with missing values. Protein quantification and statistical significance analysis were performed using a two-way Student’s t-test. Proteins that were quantified with a fold change (FC) of >1.5 and a significance value of *p* < 0.05 after comparing the two groups were designated as DEPs, while log2 (FC) was used for further analysis.

### 2.7 Enzyme-linked immunosorbent assay

The plasma B cell activating factors (BAFF), IL-4, IL-13, IL-17A, IL-22, and INF-γ concentrations were determined using ELISA kits supplied by eBioscience (San Diego, CA, United States) according to the instructions of the manufacturer. The exosomal protein ceruloplasmin (CP), cartilage oligomeric matrix protein (COMP), insulin-like growth factor-binding protein 2 (IGFBP2), ficolin-2 (FCN2), and lipopolysaccharide-binding protein (LBP) concentrations were determined using ELISA kits supplied by EIAab Science Inc. (Wuhan, China) according to the instructions of the manufacturer.

### 2.8 Clinical parameter analysis

All clinical parameters were obtained from the clinical laboratory according to standard operating procedures. The tear flow rate was evaluated using Schirmer’s test, with a positive result indicating that the length of the moisture on the filter paper was less than 5 mm per min (≤5 mm/5 min). The unstimulated salivary flow was determined according to methods described in previous studies, with a positive result indicating that the amount of saliva per 15 min was less than 1.5 ml (≤1.5ml/15 min).

### 2.9 Bioinformatic analysis

The information of identified proteins was obtained from the UniProt-GOA database (http://www.ebi.ac.uk/GOA/). Both Gene Ontology (GO) analysis and Kyoto Encyclopedia of Genes and Genomes (KEGG) pathway analysis were conducted by DAVID online database (https://david.ncifcrf.gov/). Main steps are as followed: Firstly, open website and click “functional annotation”, paste target gene in “Enter Gene list” item, and select “Official_Gene_Symbol” in “Select Identifiter” item, select “Gene list” in “list type” item, and click “use” in select “*Homo sapiens*” item and the system will automatically perform enrichment analysis. Secondly, click the “Gene_Ontology” drop-down option, select “GO term_BP_direct,” “GO term_CC_direct” and “GOterm_MF_direct” respectively, and click “Function Annotation” to get the final GO enrichment result, and to save in “up_GO. txt” file for visual analysis. Thirdly, click the “Pathway” drop-down option, select “KEGG pathway” to get the final KEGG enrichment result, and to save in “KEGG_pathway. txt” file for visual analysis. Finally, the top 20 items are used for go visual analysis and the top 15 items are used for KEGG visual analysis.

### 2.10 Statistical analysis

The data were shown as the median with the interquartile range or standard deviation. The differences in the clinical parameters and plasma cytokines before and after treatment were evaluated via a paired *t*-test. The exosomal proteins before and after treatment that needed verification via ELISA were evaluated using an unpaired *t*-test. A value of *p* < 0.05 was considered statistically significant. All statistical analyses were performed using GraphPad Prism V9 software (GraphPad Software, San Diego, CA, United States).

## 3 Results

### 3.1 The effect of modified zengye decoction on the clinical symptoms and laboratory examination parameters of Sjögren's syndrome patients

**FIGURE 1:**
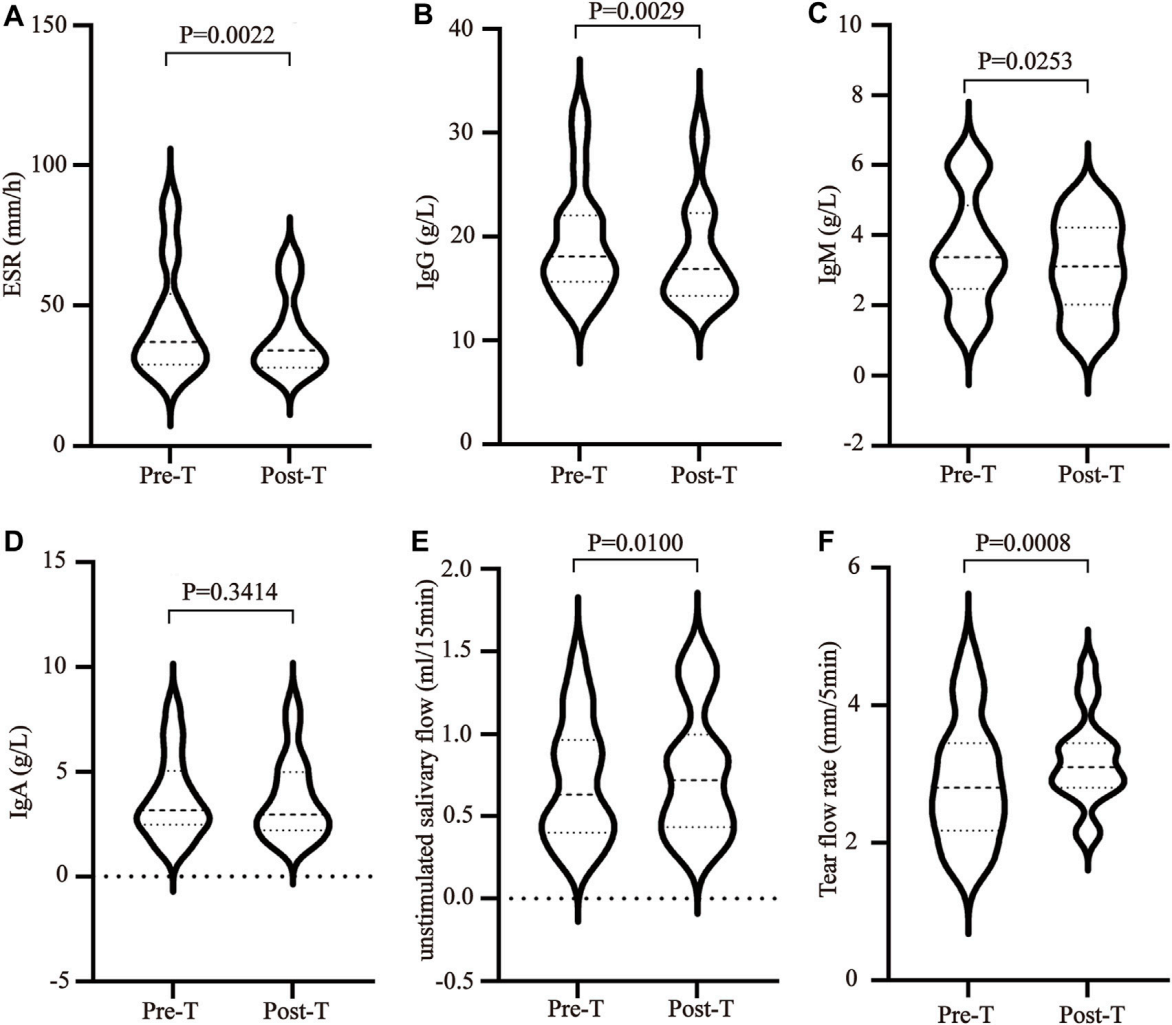
The influence of MZD on SS laboratory test subjects. After the SS patients were exposed to continuous MZD intervention for 2 weeks, the differences before and after treatment were compared via a paired *t*-test. **(A)**The ESR levels. The **(B)** IgG, **(C)** IgM, and **(D)** IgA concentrations. **(E)** The salivary flow rate. **(F)** The tear flow rate.

MZD can effectively improve the clinical symptoms of SS. Effective treatment rates of 99.9% were evident for a dry tongue, dry eyes, dry stool, and dry skin. The effective treatment rates for parotid gland swelling, hand and foot heart heat, low fever, joint pain, and rash were 84.62%, 81.82%, 75.00%, 68.75%, and 62.50%, respectively (Table 1). In addition, MZD also significantly affected the laboratory examination parameters. Compared with pre-treatment, the erythrocyte sedimentation rate (ESR) of the patients after treatment was reduced considerably, *p* = 0.0022 (Figure 1A), while the plasma immune IgG and IgM content decreased substantially, with *p*-values of 0.0029 (Figure 1B) and 0.0253 (Figure 1C), respectively. However, no significant changes were evident in IgA (Figure 1D). A considerable increase was apparent in both the salivary flow rate, *p* = 0.01 (Figure 1E), and tear flow rate, *p* = 0.0008 (Figure 1F).

**TABLE 1:** MZD improves the main clinical symptoms of SS patients.

Clinical symptoms	Number of cases(n)	Number of improvement cases(n)	Efficiency (%)
Dry mouth and tongue	18	18	100.00
Dry eyes	18	18	100.00
Dry skin	16	16	100.00
Dry stool	15	15	100.00
Feverish palms and soles	11	9	81.82
Enlargement of parotid gland	13	11	84.62
Rash	8	5	62.50
Arthronalgia	16	11	68.75
Low-grade fever	8	6	75.00

### 3.2 The effect of modified zengye decoction on the plasma cytokines in Sjögren's syndrome

**FIGURE 2:**
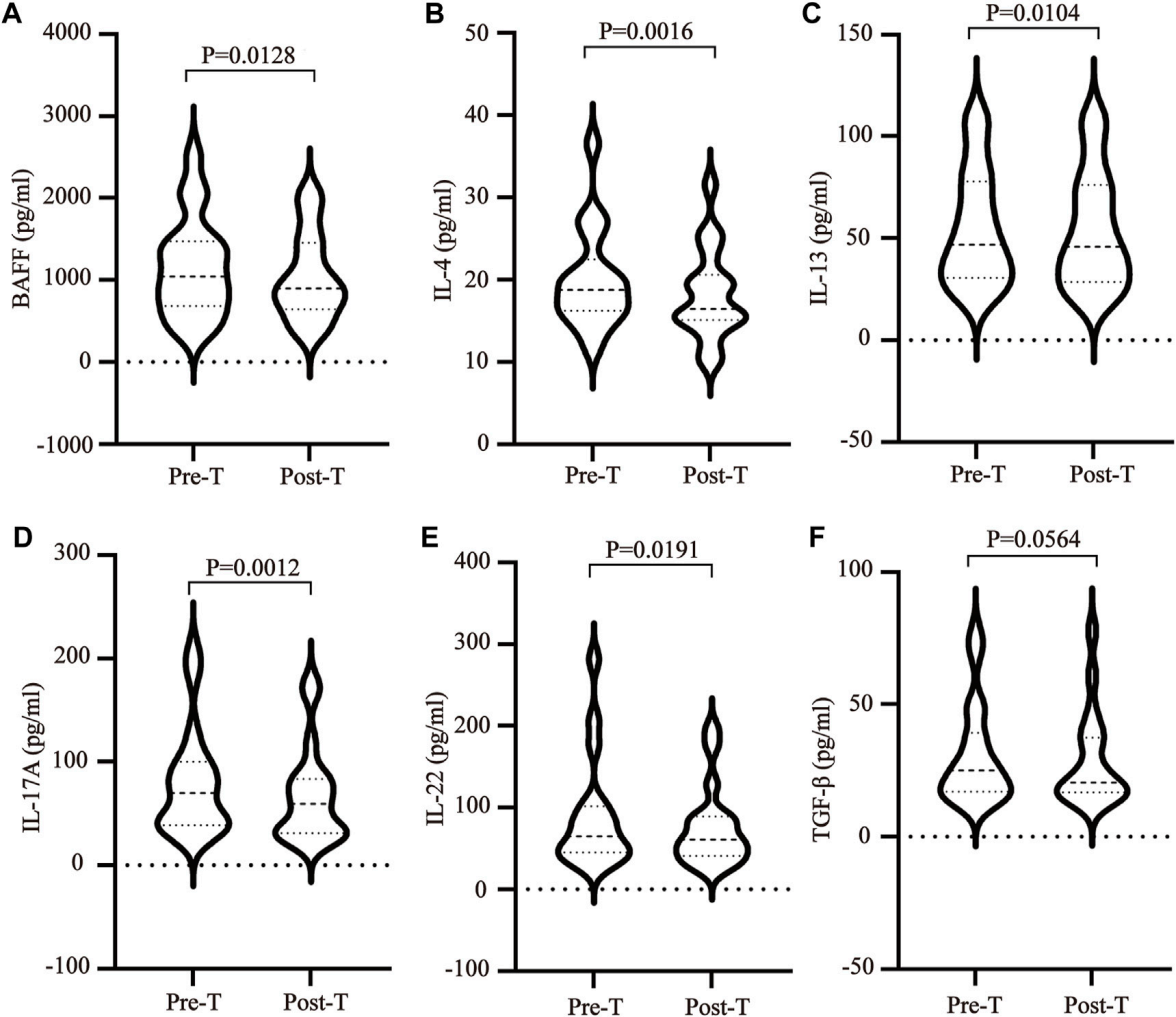
The effect of MZD on the plasma cytokines. A paired *t*-test was used to compare the plasma cytokine content before and after treatment. The **(A)**BAFF **(B)** IL-4, **(C)** IL-13, **(D)** IL-17A, **(E)** IL-22, and **(F)** TGF-β plasma concentrations before and after treatment.

The imbalance between T helper cells (Th cells) subset differentiation and its related cytokines plays an important role in the pathological process of SS. To clarify the effect of MZD on the cell subset differentiation and cytokines, the changes in the plasma Treg cytokines (TGF-β), Th2 cytokines (IL-4 and IL-13), Th17 cytokines (IL-17A and IL-22), and BAFF were examined before and after MZD treatment. The results showed that the BAFF (Figure 2A), IL-4 (Figure 2B), IL-13 (Figure 2C), IL-17A (Figure 2D), and IL-22 (Figure 2E) plasma content decreased significantly after MZD treatment, with *p*-values of 0.0128, 0.0016, 0.0104, 0.0012, and 0.0191, respectively. The TGF-β content displayed no significant changes after treatment (Figure 2F). This suggests that although MZD can substantially downregulate the Th2 and Th17 cytokine levels, it does not significantly affect the TGF-βcontent.

### 3.3The differentially expressed protein levels before and after modified zengye decoction treatment of the Sjögren's syndrome patients

**FIGURE 3:**
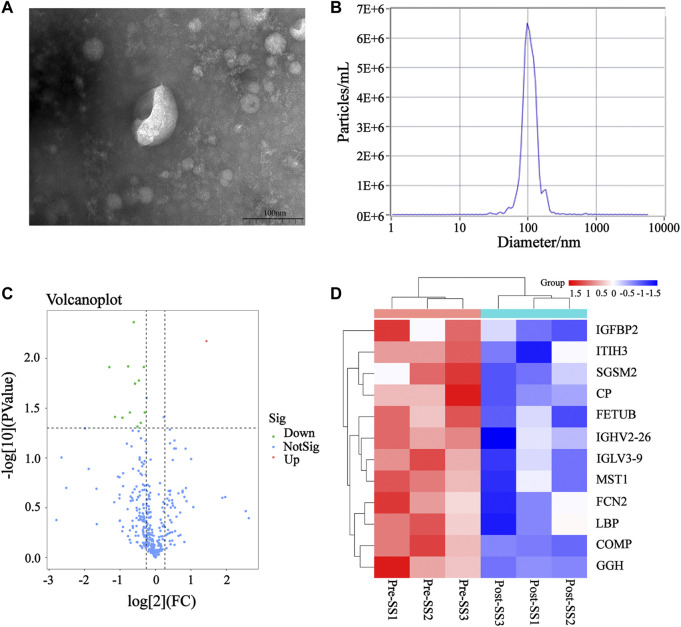
The DEPs in the plasma exosomes before and after MZD treatment of SS. **(A)** The TEM images of the mixed exosomes are shown at 110,000×magnification. Scale bar = 100 nm. **(B)**The size distribution of the exosomes was analyzed *via* NTA. **(C)** A volcano map of the 13 DEPs in the plasma exosomes. **(D)** A clustering heat map of the 12 downregulated DEPs. The expression of each protein is illustrated in red and green to indicate high and low expression, respectively.

The plasma exosomes were isolated using UC, while the exosomal DEPs before and after MZD treatment were identified via label-free proteomics. Transmission electron microscopy (TEM) showed typical cup-shaped vesicles (Figure 3A), while Nanoparticle tracking analysis (NTA) indicated that these vesicles were approximately 100 nm in diameter (Figure 3B). A quantitative ratio exceeding 1.5 was considered upregulation, while a ratio below 0.667 was considered downregulation. A comparison between the results before and after MZD treatment indicated that 13 DEPs were identified in the plasma exosomes via label-free proteomic analysis, one of which was an upregulated protein, while twelve were downregulated proteins, as shown in the DEP volcano map (Figure 3C) and downregulated protein clustering heat map (Figure 3D). The detailed data of the upregulated and downregulated proteins are shown in Table2.

**TABLE 2:** The plasma exosomalDEPs before and after MZD treatment.

Accession	Gene symbol	Protein name	FC	P
P18065	IGFBP2	Insulin-like growth factor-binding protein 2	0.4061	0.0122
Q06033	ITIH3	Inter-alpha-trypsin inhibitor heavy chain H3	0.6543	0.0043
O43147	SGSM2	Small G protein signaling modulator 2	0.7490	0.0444
P00450	CP	Ceruloplasmin	0.7950	0.0122
Q9UGM5	FETUB	Fetuin-B	0.7235	0.0167
A0A0B4J1V2	IGHV2-26	Immunoglobulin heavy variable 2-26	0.7036	0.0485
P24593	IGFBP5	Insulin-like growth factor-binding protein 5	2.7084	0.0067
A0A075B6K5	IGLV3-9	Immunoglobulin lambda variable 3-9	0.5856	0.0121
P26927	MST1	Hepatocyte growth factor-like protein	0.8119	0.0350
Q15485	FCN2	Ficolin-2	0.5233	0.0394
P18428	LBP	Lipopolysaccharide-binding protein	0.6697	0.0180
P49747	COMP	Cartilage oligomeric matrix protein	0.4523	0.0388
Q92820	GGH	Gamma-glutamyl hydrolase	0.6058	0.0349

### 3.4 Analysis of the biological information of the exosomal downregulated differentially expressed proteins

**FIGURE 4:**
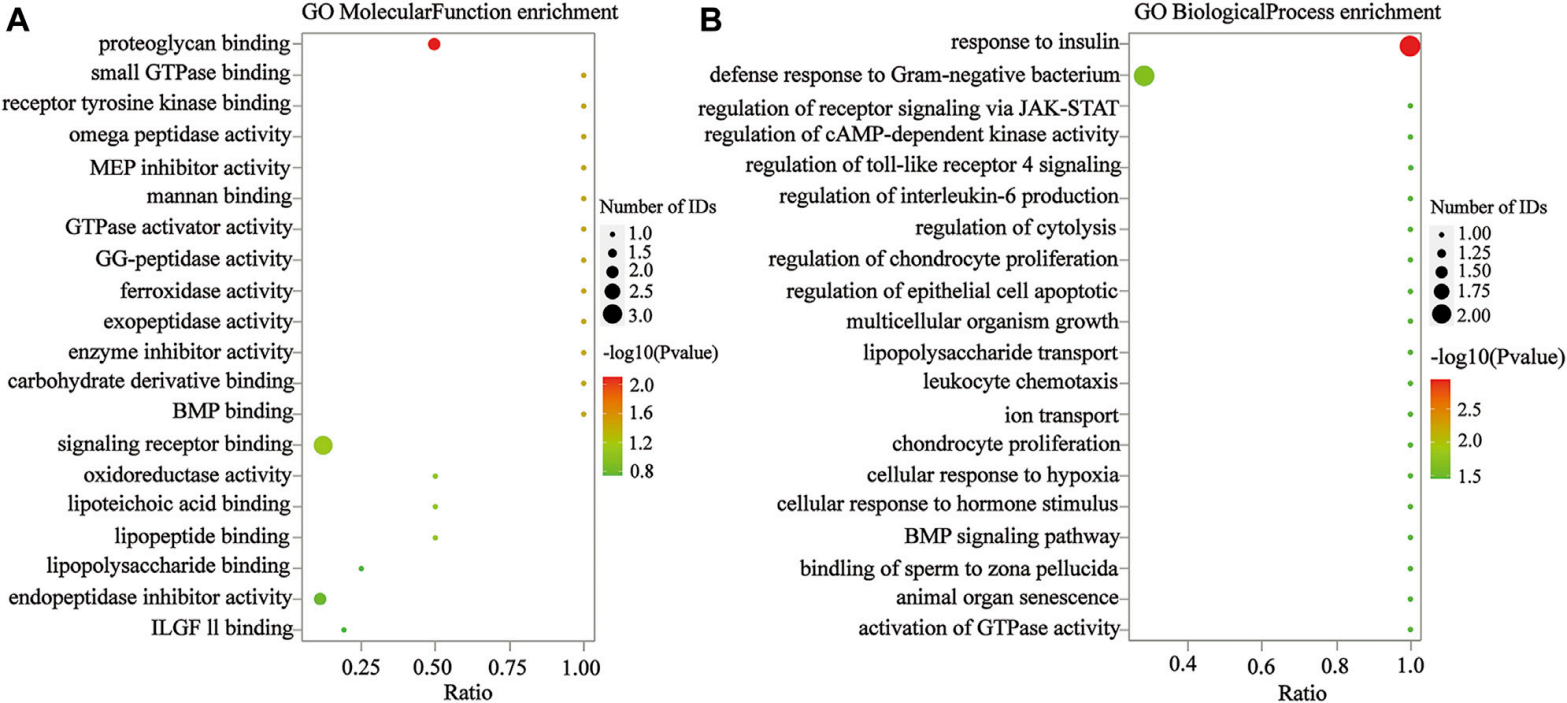
The GO analysis of the downregulated DEPs in the plasma exosomes. **(A)** The MF enrichment analysis. **(B)** The BP enrichment analysis. The circle size represents the number of genes enriched in the GO entries. A larger circle indicates more gene enrichment in this GO entry. The color of the circle represents the significance of the enrichment. The redder the circle, the more significant the DEP enrichment on this GO entry.

To confirm that the therapeutic effect of MZD on SS is related to plasma exosomal protein reduction, this study used bioinformatics to analyze the biological functions of the downregulated proteins after MZD treatment. As shown in Figure 4A and Supplementary Table S1, the results of the GO analysis (BP) indicated that the downregulated proteins were significantly involved in the response to insulin (*p* = 0.0011) and the defense response to Gram-negative bacteria (*p* = 0.0217). The insulin response may be related to the dryness symptoms caused by SS, while the defense response to Gram-negative bacteria may be associated with SS infection. As shown in Figure 4B and Supplementary Table S2, the GO analysis (MF) results indicated that the downregulated proteins were significantly enriched in proteoglycan binding (*p* = 0.0079), which may also be associated with SS infection. As shown in Figure 4C and Supplementary Table S3, the KEGG pathway analysis showed that the downregulated proteins were completely enriched in porphyrin metabolism (*p* = 0.0280), folate biosynthesis (*p* = 0.0280), antifolate resistance (*p* = 0.0280), the NF-κB signaling pathway (*p* = 0.0454), the toll-like receptor signaling pathway (*p* = 0.0454), and ferroptosis (*p* = 0.0454). Porphyrin metabolism and folate biosynthesis may be related to the bone and joint injury caused by SS, while the NF-κB and Toll-like receiver signaling pathways may be associated with SS infection. These results suggest that the exosomal proteins downregulated by MZD are closely related to the SS pathological mechanism.

**FIGURE 5:**
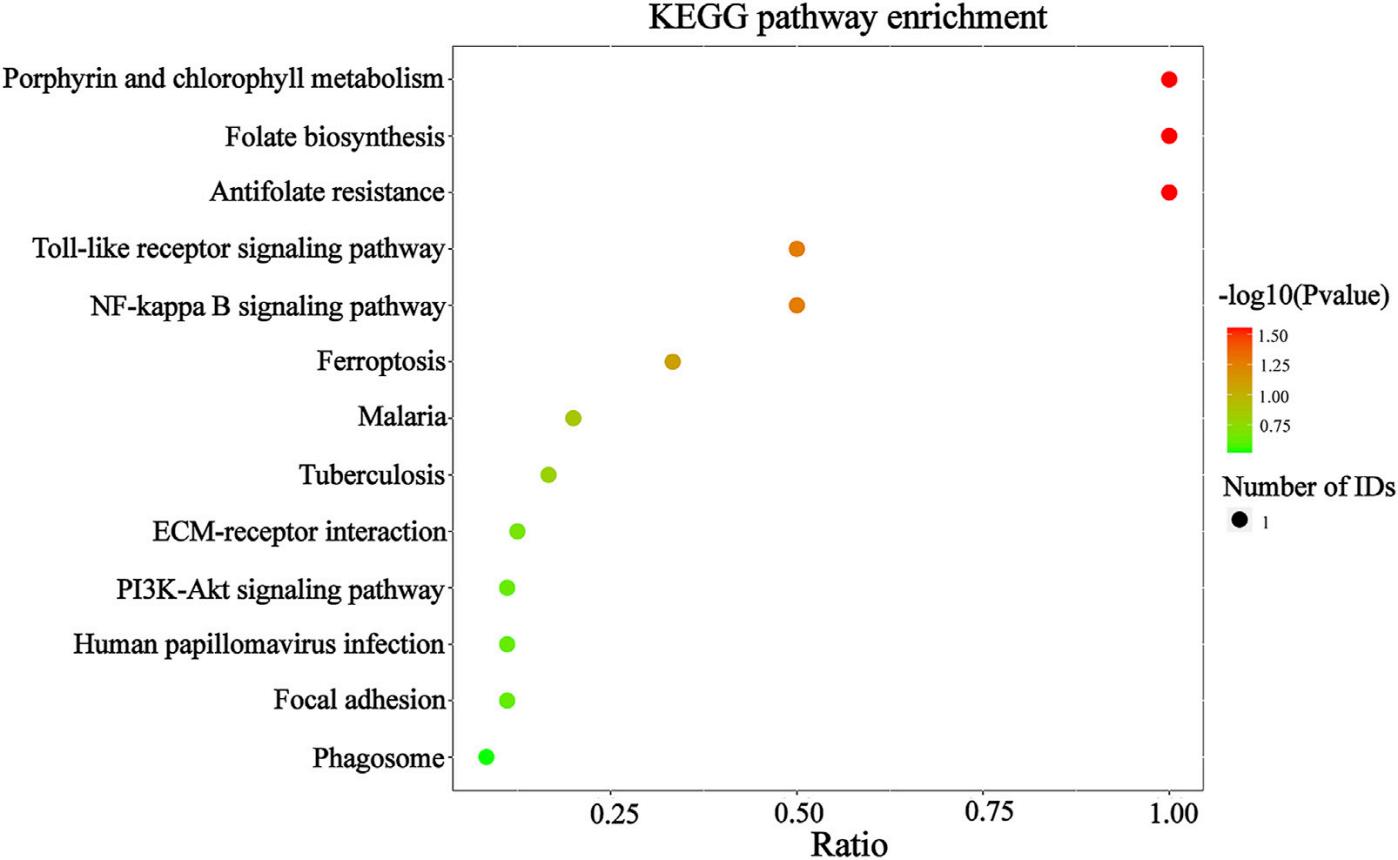
The KEGG analysis of the downregulated DEPs in the plasma exosomes. The color of the circle represents the significance of the enrichment. The redder the circle, the more significant the DEP enrichment in this entry of the KEGG pathway. The 13 enriched KEGG pathway terms representing the downregulated DEPs are displayed.

### 3.5 The validation of the downregulated exosomal differentially expressed proteins (related to modified zengye decoction efficacy

**FIGURE 6:**
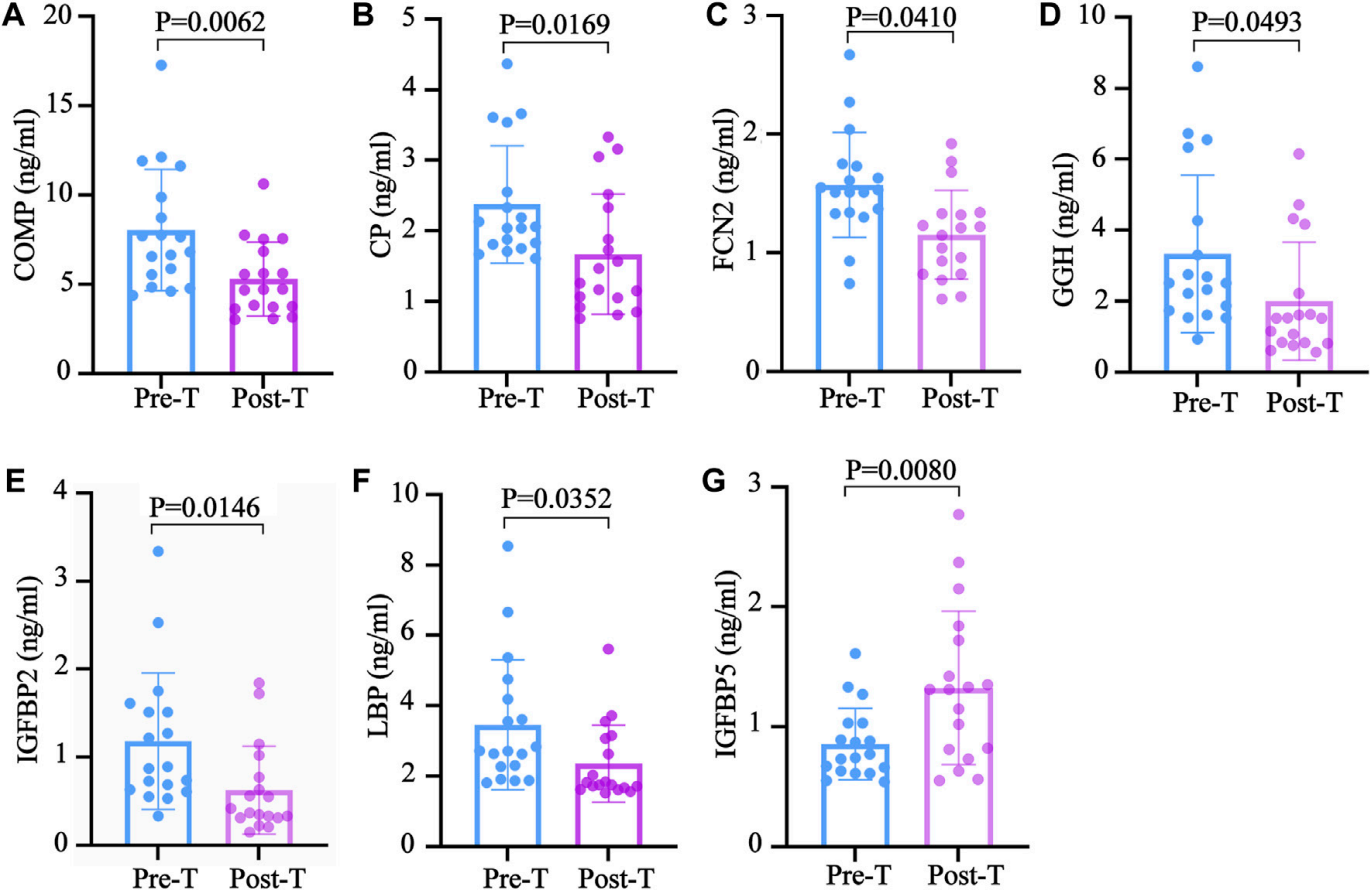
The validation of the downregulated exosomal proteins associated with SS. The ELISA test results are shown using a bar graph, expressed as mean ± standard deviation. An unpaired *t*-test was used to compare the changes before and after treatment. The **(A)** COMP **(B)** CP, **(C)** FCN2 **(D)** GGH, **(E)** IGFBP2 **(F)** LBP, and **(G)** IGFBP5 concentrations in the plasma before and after treatment.

After examining the GO and KEGG analyses results, this study speculates that the exosomal proteins downregulated by MZD may include COMP,CP,FCN2,GGH, IGFBP2 and LBP. Detailed information about the BPs, MFs and signaling pathways involved in these proteins are provided in Supplementary Tables S1–S3. The ELISA results of these exosomal proteins were consistent with the label-free proteomics findings. After treatment, the COMP, CP, FCN2, GGH, IGFBP2, and LBP expression levels were significantly decreased. The detailed detection results are shown in Figures 6A–F, respectively.

## 4 Discussion

Although genetic, environmental, and viral factors play an important role in the onset of SS, the exact mechanism driving this disease remains unclear (Roszkowska et al., 2021). From a clinical and pathological perspective, the primary hallmark of this disease is exocrine dysfunction, including salivary and lacrimal gland malfunction (Negrini et al., 2022). Therefore, the primary clinical manifestations of SS are xerostomia and xeropthalmia, severely impairing the quality of life of patients (Vehof et al., 2020). Treatment for the disease remains largely palliative, focusing on stimulating the remaining secretion of exocrine glands or replacing exocrine fluid of saliva and tears (Chowdhury et al., 2021). However, the existing treatments are primarily unsuccessful in improving symptoms and disease progression (Fox et al., 2021). Therefore, it is necessary to find new methods to improve the therapeutic effect of SS.

Since traditional Chinese medicine compounds can effectively alleviate SS symptoms (Chen et al., 2021; Hu et al., 2014; Shao et al., 2021), many patients in China opt for this treatment (Hou and Xue, 2020). This study showed that MZD could effectively improve the clinical symptoms of SS patients receiving continuous treatment for 2 weeks, including a dry tongue, dry eyes, dry skin, and dry stool. It also exhibited an excellent therapeutic effect on joint pain, parotid gland swelling, and rash. Similarly, the laboratory test results supported the therapeutic effect of traditional Chinese medicine on SS, which was mainly reflected in the significant increase in the salivary flow rate, the substantially lower ESR, and the considerable decrease in the content of the plasma immune indexes, IgG and IgM, after MZD treatment. These results suggest that MZD or other traditional Chinese medicine may provide a new strategy for SS treatment.

The main pathological feature of SS is the infiltration of the exocrine glands by a large number of lymphocytes. Some studies have shown that the abnormal differentiation of Th2 cells and Th17 cells and their cytokines play an important role in the occurrence of SS (Qi et al., 2018; Huang et al., 2020). The plasma content of Th2 cytokines, IL-4 and IL-13, and Th17 cytokines, IL-17A and IL-22, decreased significantly after MZD treatment, suggesting that MZD inhibited Th2 and Th17 activation and the mediated inflammatory response. These results were consistent with previously published studies (Wang et al., 2007; Lee et al., 2021), which showed that MZD could effectively inhibit the activation of Th2 and Th17 cells and the production of corresponding cytokines. Furthermore, traditional Chinese medicine also inhibited BAFF production. Since BAFF activates B cells, its inhibition may be essential for the downregulation of the IgG and IgM antibody levels in traditional Chinese medicine. In addition, MZD also affected the TGF-β. Tregs are inhibitory T cells that produce TGF- *β* that can inhibit the inflammatory effect of Th17 cells. The results showed that Chinese herbal medicine had no significant impact on the TGF-β plasma content. Although the TGF-β level decreased after treatment, no significant differences were evident before and after treatment. This study indicated that the therapeutic effect of traditional Chinese medicine on SS may be related to the inhibition of Th2 and Th17 activation and the corresponding cytokine reduction without damaging the inhibitory function of Treg cells.

Exosomes carry several proteins, nucleic acids, and lipids that extensively participate in transmitting information between cells. Various studies have shown that exosomes play a crucial role in the occurrence of autoimmune diseases, tumors, and cardiovascular diseases (Tan et al., 2016). Exosomes can reportedly regulate the differentiation of Th2 cells and Th17 cells. Therefore, it is speculated that the therapeutic effect of MZD on SS may be related to decreased plasma exosome protein. Label-free proteomic analysis showed the presence of13 DEPs in the plasma exosomes before and after MZD intervention, of which 12 were downregulated. The downregulated proteins were involved in several BFs, including the insulin response, the Gram-negative bacterial defense response, and proteoglycan binding. The insulin response is closely related to a dry mouth in diabetic patients (Sundaram et al., 2020). This study also speculates that the dry mouth symptoms in SS patients are also related to the insulin response. The alleviating effect of traditional Chinese medicine on dryness may also be related to the downregulation of the insulin response. The defense response to Gram-negative bacteria and proteoglycan binding plays a vital role in the pathological processes of infection, consistent with the cause of SS infection. This suggests that the therapeutic effect of Chinese medicine on SS may be related to the inhibition of SS infection. The KEGG signaling pathway enrichment also showed similar results. The downregulated exosome proteins were mainly involved in the NF-κB and TLR4 pathways and porphyrin metabolism. The NF-κB and TLR4 signaling pathways are crucial in activating inflammation, further suggesting that the therapeutic effect of traditional Chinese medicine is related to the inhibition of SS infection. Porphyrin metabolism and folic acid synthesis are involved in almost all autoimmune diseases, and the influence of traditional Chinese medicine on the metabolic pathway may also highlight its efficacy. To ensure the reliability of the proteomic results, this study verified the downregulated exosomal proteins, namely COMP, CP, FCN2, GGH, IGFBP2, and LBP, involved in the BPs. The changes in these proteins before and after treatment were consistent with the proteomic results. This study suggests that the therapeutic MZD mechanism in SS may be related to the reduced expression of the exosomal proteins involved in the insulin response, porphyrin metabolism, and the TLR4/NF-κB pathway.

Therefore, this research confirms that MZD can effectively relieve the clinical symptoms of SS and verifies that its action mechanism may be related to exosomal protein reduction. Furthermore, the MZD efficacy verification results provided evidence for the role of Th cell subset differentiation, corresponding cytokines, and exosomal proteins in SS. However, this research presents some limitations. First, this study does not sufficiently highlight the multi-target therapeutic effect of the Chinese herbal formula and lacks a single chemical component analysis of MZD efficacy. Second, the therapeutic mechanism of MZD inSS is only elucidated from the perspective of exosomal proteins, while other exosomal content that may be involved, such as microRNA and IncRNA, is not analyzed. Third, the sample size is small. Currently, SS treatment mainly combines Western and traditional Chinese medicine. Few patients are willing to accept traditional Chinese medicine alone, preventing the use of large samples for clinical intervention studies.

## Data Availability

The data presented in the study are deposited in the ProteomeXchange Consortium *via* the PRIDE partner respository with the dataset identifier PXD033627.

## Abbreviations

American College of Rheumatology (ACR); ammonium bicarbonate (ABC); automatic gain control (AGC); biological process (BP); cartilage oligomeric matrix protein (COMP); cellular compartment (CC); ceruloplasmin (CP); differentially expressed proteins (DEPs); European League Against Rheumatism (EULAR); factor-binding protein 2 (IGFBP2); ficolin-2 (FCN2); false discovery rate (FDR); fold change (FC); Gene Ontology (GO); higher-energy collisional dissociation (HCD); Kyoto Encyclopedia of Genes and Genomes (KEGG); label-free quantification (LFQ); lipopolysaccharide-binding protein (LBP); Modified Zengye Decoction (MZD); molecular function (MF); peptide-spectrum match (PSM); primary Sjögren’s syndrome (pSS); Traditional Chinese medicine (TCM); ultracentrifugation (UC); dithiothreitol (DTT).
