# Supplementary material for: Therapeutic effect of modified zengye decoction on primary Sjogren’s syndrome and its effect on plasma exosomal proteins
Source: Front Pharmacol. 2022 Aug 26;13:930638. doi: 10.3389/fphar.2022.930638 (PMC9462528; doi:10.3389/fphar.2022.930638)
Supplement: Supplementary file 1 [file Table1.docx]

Supplementary Table 1 Detailed information of down-regulated exosomal proteins GO analysis(MF) after MZD treatment

| **TermID** | **Term** | **Pvalue** | **Enrichment** | **Gene-symbol** |
| --- | --- | --- | --- | --- |
| GO:0053395 | proteoglycan binding | 0.0079 | 2.1043 | FCN2,COMP |
| GO:0008191 | MEP inhibitor activity | 0.0386 | 1.5136 | FETUB |
| GO:2001065 | mannan binding | 0.0386 | 1.5136 | FCN2 |
| GO:0030971 | receptor tyrosine kinase binding | 0.0386 | 1.5136 | MST1 |
| GO:0034722 | GG-peptidase activity | 0.0386 | 1.5136 | GGH |
| GO:0008238 | exopeptidase activity | 0.0386 | 1.5136 | GGH |
| GO:0004857 | enzyme inhibitor activity | 0.0386 | 1.5136 | FETUB |
| GO:0031267 | small GTPase binding | 0.0386 | 1.5136 | SGSM2 |
| GO:0004322 | ferroxidase activity | 0.0386 | 1.5136 | CP |
| GO:0036122 | BMP binding | 0.0386 | 1.5136 | COMP |
| GO:0097367 | carbohydrate derivative binding | 0.0386 | 1.5136 | FCN2 |
| GO:0005096 | GTPase activator activity | 0.0386 | 1.5136 | SGSM2 |
| GO:0008242 | omega peptidase activity | 0.0386 | 1.5136 | GGH |
| GO:0005102 | signaling receptor binding | 0.0757 | 1.1210 | IGFBP2,FCN2,LBP |
| GO:0016491 | oxidoreductase activity | 0.0758 | 1.1203 | CP |
| GO:0070891 | lipoteichoic acid binding | 0.0758 | 1.1203 | LBP |
| GO:0071723 | lipopeptide binding | 0.0758 | 1.1203 | LBP |
| GO:0001530 | lipopolysaccharide binding | 0.1463 | 0.8348 | LBP |
| GO:0004866 | endopeptidase inhibitor activity | 0.1618 | 0.7909 | ITIH3,FETUB |
| GO:0031995 | ILGF II binding | 0.1797 | 0.7455 | IGFBP2 |
