# Supplementary material for: Therapeutic effect of modified zengye decoction on primary Sjogren’s syndrome and its effect on plasma exosomal proteins
Source: Front Pharmacol. 2022 Aug 26;13:930638. doi: 10.3389/fphar.2022.930638 (PMC9462528; doi:10.3389/fphar.2022.930638)
Supplement: Supplementary file 2 [file Table2.docx]

Supplementary Table 2 Detailed information of down-regulated exosomal proteins GO analysis(BP) after MZD treatment

| **TermID** | **Term** | **Pvalue** | **Enrichment** | **Gene-symbol** |
| --- | --- | --- | --- | --- |
| GO:0032868 | response to insulin | 0.0011 | 2.9437 | IGFBP2,GGH |
| GO:0050829 | defense response to Gram-negative bacterium | 0.0217 | 1.6645 | FCN2,LBP |
| GO:1904036 | regulation of epithelial cell apoptotic | 0.0352 | 1.5357 | MST1 |
| GO:0007339 | binding of sperm to zona pellucida | 0.0352 | 1.5357 | FETUB |
| GO:0046425 | regulation of receptor signaling via JAK-STAT | 0.0352 | 1.5357 | MST1 |
| GO:0032755 | regulation of interleukin-6 production | 0.0352 | 1.5357 | LBP |
| GO:0035264 | multicellular organism growth | 0.0352 | 1.5357 | COMP |
| GO:0010260 | animal organ senescence | 0.0352 | 1.5357 | COMP |
| GO:0030509 | BMP signaling pathway | 0.0352 | 1.5357 | COMP |
| GO:0034145 | regulation of toll-like receptor 4 signaling | 0.0352 | 1.5357 | LBP |
| GO:0090630 | activation of GTPase activity | 0.0352 | 1.5357 | SGSM2 |
| GO:0071456 | cellular response to hypoxia | 0.0352 | 1.5357 | MST1 |
| GO:0045919 | regulation of cytolysis | 0.0352 | 1.5357 | LBP |
| GO:0032870 | cellular response to hormone stimulus | 0.0352 | 1.5357 | IGFBP2 |
| GO:0006811 | ion transport | 0.0352 | 1.4536 | CP |
| GO:1902732 | regulation of chondrocyte proliferation | 0.0352 | 1.4536 | COMP |
| GO:0002232 | leukocyte chemotaxis | 0.0352 | 1.4536 | LBP |
| GO:0015920 | lipopolysaccharide transport | 0.0352 | 1.4536 | LBP |
| GO:2000479 | regulation of cAMP-dependent kinase activity | 0.0352 | 1.4536 | MST1 |
| GO:0035988 | chondrocyte proliferation | 0.0352 | 1.4536 | COMP |
