# Supplementary material for: Therapeutic effect of modified zengye decoction on primary Sjogren’s syndrome and its effect on plasma exosomal proteins
Source: Front Pharmacol. 2022 Aug 26;13:930638. doi: 10.3389/fphar.2022.930638 (PMC9462528; doi:10.3389/fphar.2022.930638)
Supplement: Supplementary file 3 [file Table3.docx]

Supplementary Table 3 Detailed information of down-regulated exosomal proteins KEGG analysis after MZD treatment

| **TermID** | **Term** | **Pvalue** | **Enrichment** | **Gene-symbol** |
| --- | --- | --- | --- | --- |
| path:hsa00860 | Porphyrin and chlorophyll metabolism | 0.0280 | 1.5533 | CP |
| path:hsa00790 | Folate biosynthesis | 0.0280 | 1.5533 | GGH |
| path:hsa01523 | Antifolate resistance | 0.0280 | 1.5533 | GGH |
| path:hsa04064 | NF-kappa B signaling pathway | 0.0454 | 1.2569 | LBP |
| path:hsa04620 | Toll-like receptor signaling pathway | 0.0454 | 1.2569 | LBP |
| path:hsa04216 | Ferroptosis | 0.0472 | 1.0854 | CP |
| path:hsa05144 | Malaria | 0.1340 | 0.8728 | COMP |
| path:hsa05152 | Tuberculosis | 0.1591 | 0.7982 | LBP |
| path:hsa04512 | ECM-receptor interaction | 0.2077 | 0.6826 | COMP |
| path:hsa04151 | PI3K-Akt signaling pathway | 0.2312 | 0.6361 | COMP |
| path:hsa04510 | Focal adhesion | 0.2312 | 0.6361 | COMP |
| path:hsa05165 | Human papillomavirus infection | 0.2312 | 0.6361 | COMP |
| path:hsa04145 | Phagosome | 0.2985 | 0.5251 | COMP |
